# Anti-tumoral effect of desmethylclomipramine in lung cancer stem cells

**DOI:** 10.18632/oncotarget.4700

**Published:** 2015-07-01

**Authors:** Lucilla Bongiorno-Borbone, Arianna Giacobbe, Mirco Compagnone, Adriana Eramo, Ruggero De Maria, Angelo Peschiaroli, Gerry Melino

**Affiliations:** ^1^ Department of Experimental Medicine and Surgery, University of Rome “Tor Vergata”, Via Montpellier, Rome, Italy; ^2^ Department of Hematology, Oncology and Molecular Medicine, Istituto Superiore di Sanità, Rome, Italy; ^3^ Regina Elena National Cancer Institute, Rome, Italy; ^4^ Institute of Cell Biology and Neurobiology, CNR, Rome, Italy; ^5^ Medical Research Council, Toxicology Unit, Hodgkin Building, Leicester University, Leicester, United Kingdom

**Keywords:** non-small lung cancer stem cells, Itch inhibitor, DCMI, chemoresistance

## Abstract

Lung cancer is the most feared of all cancers because of its heterogeneity and resistance to available treatments. Cancer stem cells (CSCs) are the cell population responsible for lung cancer chemoresistance and are a very good model for testing new targeted therapies. Clomipramine is an FDA-approved antidepressant drug, able to inhibit in vitro the E3 ubiquitin ligase Itch and potentiate the pro-apoptotic effects of DNA damaging induced agents in several cancer cell lines. Here, we investigated the potential therapeutic effect of desmethylclomipramine (DCMI), the active metabolite of Clomipramine, on the CSCs homeostasis. We show that DCMI inhibits lung CSCs growth, decreases their stemness potential and increases the cytotoxic effect of conventional chemotherapeutic drugs. Being DCMI an inhibitor of the E3 ubiquitin ligase Itch, we also verified the effect of Itch deregulation on CSCs survival. We found that the siRNA-mediated depletion of Itch induces similar anti-proliferative effects on lung CSCs, suggesting that DCMI might exert its effect, at least in part, by inhibiting Itch. Notably, Itch expression is a negative prognostic factor in two primary lung tumors datasets, supporting the potential clinical relevance of Itch inhibition to circumvent drug resistance in the treatment of lung cancer.

## INTRODUCTION

Lung cancer is the leading cause of cancer-related deaths worldwide [1, 2]. Most patients relapse after surgery and require medical treatment like patients diagnosed with a metastatic disease. Despite recent advances in treatment of subsets of patients, the vast majority of patients receive chemotherapy and soon become chemoresistant [3]. This is the reason why the overall 5-year survival of patients diagnosed with lung cancer is less than 15% [4]. Chemotherapy predominantly kills the drug-sensitive cells, leaving behind a heterogeneous population of resistant cells that gradually expand to produce a chemoresistant tumor. Recent studies have demonstrated that a specialized population of tumor cells named cancer stem cells (CSCs) or tumor-initiating cells is thought to be responsible for tumor initiation, progression and resistance to therapy [5]. We have identified lung CSCs and developed a technology for *in vitro* and *in vivo* expansion and characterization, which allow us testing and preclinical validation of new targeted therapies [6, 7].

A current strategy to enhance the efficacy of anticancer therapy involves the usage of drugs deregulating autophagic processes. Autophagy is a conserved lysosome-mediated process, which degrades cellular organelles and macromolecules, allowing the recycling of bioenergetics components in order to favour the survival of cells in response to diverse stress like starvation, hypoxia and endoplasmatic reticulum stress [8, 9]. Besides its role in the regulation of several biological processes, autophagy is also known to be closely involved in many human diseases, including cancer [9, 10]. However, the role of autophagy in tumor progression is controversial and may depend on various factors, such as the cancer type, the development stage and the genetic background [11-14]. Currently, several drugs targeting autophagy process has been tested and some of them are in clinical trials [15, 16]. Clomipramine is an FDA-approved drug generally used for treatment of obsessive-compulsive disorders [17, 18]. It has a long-standing record with good subject tolerance. Besides its function as noradrenergic and serotonergic reuptake inhibitor, clomipramine acts as a regulator of autophagy [19, 20]. Treating cells with clomipramine or its active metabolite desmethylclomipramine (DCMI) induces the blockade of the autophagic flux, as revealed by the increase of authophagosomal markers and a concomitant blockade of the degradation of autophagic cargo, such as p62. Importantly, DCMI increases the pro-apoptotic effects of conventional chemotherapic drugs in several cancer cell lines [21].

Recently, clomipramine has been also identified as an inhibitor of Itch, an E3 ubiquitin ligase belonging to the HECT-type family of E3 ubiquitin ligase [22]. By controlling the proteasomal-dependent degradation of a subset of target proteins, Itch regulates several important biological processes, such as apoptosis, cell growth and inflammation [23-25]. Several reports have demonstrated that the expression levels of Itch affect the apoptotic response induced by the chemotherapeutic drugs [26-28]. In details, it has been shown that Itch depletion by siRNA increases the cytotoxic effect of anti-neoplastic drugs in different cancer cell lines and the *in vivo* administration of siRNA duplex targeting Itch mRNA is effective in sensitizing pancreatic cancer to gemcitabine [29]. The pro-apoptotic effects exerted by Itch depletion are more evident in cells with no functional p53, highlighting the importance that changes in levels of Itch may play in majority of cancers, where p53 is absent or mutated.

In the present manuscript, we investigate the biological effect of DCMI on the growth properties of lung CSCs isolated from non-small-cell lung cancers (NSCLC) surgical specimens. We report that DCMI inhibits lung CSC growth, decreases their stemness potential and increases the cytotoxic effect of conventional chemotherapeutic agents. Being the DCMI an *in vitro* inhibitor of the E3 ubiquitin ligase Itch, we also analyzed the consequences of Itch downregulation on lung CSCs. Similarly to what we observed in DCMI treated lung CSCs, the siRNA-mediated depletion of Itch decreases CSCs survival in response to gemcitabine treatment, suggesting that the pro-apoptotic effects of DCMI might be exerted, at least in part, by Itch inhibition. Notably, Itch expression is a negative prognostic factor in several primary lung cancer datasets, supporting the potential clinical relevance of Itch inhibition to circumvent drug resistance in the treatment of lung cancer.

## RESULTS

### Characterization of non-small cell lung CSCs and their resistance to conventional chemotherapeutic drugs

**Figure 1:**
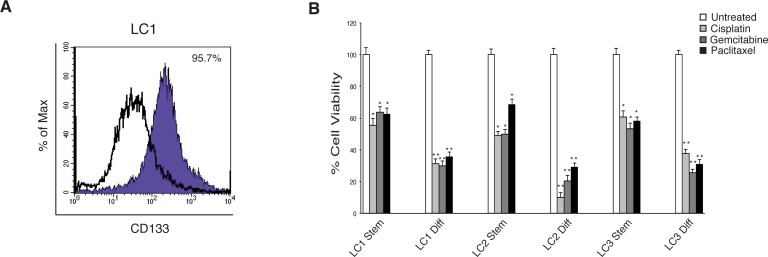
Characterization of lung CSCs and their resistance to conventional chemotherapeutic drugs **A.** Expression of CD133 detected by flow cytometry in the indicated lung CSC line (LC1). **B.** Lung CSCs (Stem) and the corresponding differentiated progeny (Diff) treated for 72 h with chemotherapeutic drugs. Cell viability was measured by Cell-Titer-Glo Assay. The experiments were performed with 2.5 μg/ml cisplatin, 50 μM gemcitabine or 30 ng/ml paclitaxel. Bars shown are the mean ± S.D. of three independent experiments. *P-value <0.05 and **P-value <0.01.

Two squamous cell carcinomas (LC1 and LC2) and one adenocarcinoma (LC3) lung CSCs were isolated from NSCLC surgical samples and characterized for the presence of common genetic alterations exhibited by lung tumors and for their ability to histologically recapitulate the tumor of origin in mice (Table 1) [7, 30]. In serum-free medium containing EGF and basic-FGF these cells grow as tumor spheroids expressing stem cell markers such as CD133. Upon serum addition the lung CSCs reduce their stemness potential, as indicated by the decreased expression of CD133 (Figure 1A).

**Table 1:** Mutation status of non-small lung CSCs used in this study

	KRAS exon 1	KRAS exon 2	p53 exon 5	p53 exon 6	p53 exon 7	p53 exon 8	EGFR exon 18	EGFR exon 19	EGFR exon 21
LC1	WT	WT	WT	Tyr220Cys	WT	WT	WT	WT	WT
LC2	WT	WT	WT	WT	WT	WT	WT	WT	WT
LC3	WT	WT	WT	WT	Gly245Cys	WT	WT	WT	WT

Generally, CSCs are characterized by an elevated resistance to the pro-apoptotic effects induced by different chemotherapeutic treatments. To test this feature in these lung CSCs, we treated lung cancer sphere-forming cells and their differentiated progeny with cisplatin, gemcitabine or paclitaxel at doses comparable with those reached in the plasma of lung cancer treated patients and measure the cell proliferation and/or cell viability by ATP assay. In contrast to the differentiated progeny, all three lung CSCs are markedly resistant to the growth arrest/apoptotic effect induced by the chemotherapeutic drugs even after a long exposure (Figure 1B). These data demonstrated that the three lung CSCs possess the expected features of chemotherapy resistance, supporting their use in the search for new therapeutic options.

### DCMI exerts a cytostatic effect in lung CSC

**Figure 2:**
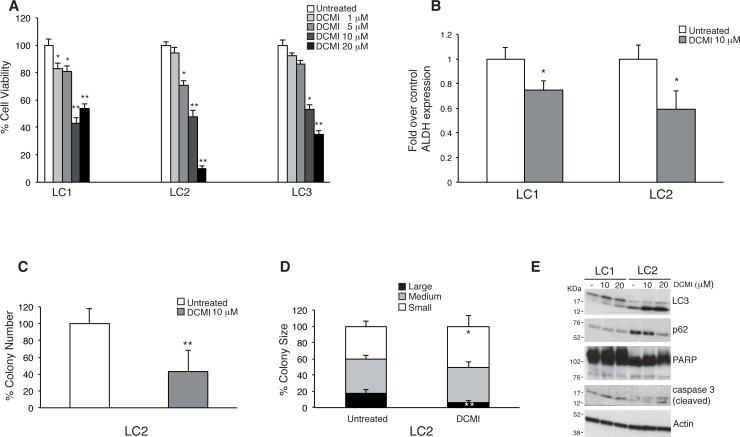
Cytostatic effect by DCMI on lung CSCs **A.** Viability of lung CSCs treated with increasing doses of DCMI (1-20 μM) for 48 h. Bars shown are the mean ± S.D. of three independent experiments. *P-value <0.05 **P-value <0.01. **B.** Fold variation of ALDEFLUOR-positive cells in DCMI treated lung CSCs as compared with vehicle-treated controls. Bars represent mean ± S.D.; *P-value <0.05 (*n* = 3). **C.** Colony formation in soft-agar culture of lung CSC LC2 plated in the presence of 10 μM DCMI. Bars represent mean ± S.D.; **P-value <0.01 (*n* = 3). **D.** Size of colonies formed in soft-agar assay by lung CSC LC2 treated as in C. Bars represent mean ± S.D.; *P-value <0.05 and **P-value <0.01 (*n* = 3). **E.** Western blot analysis of lung CSCs treated with DCMI (10 or 20 μM) for 48 hours. All whole cell extracts were analyzed by IB using antibodies to the indicated proteins.

Our group has previously demonstrated that clomipramine and its active metabolite DCMI synergize with gemcitabine in killing bladder, breast and prostate tumor cell lines [22]. To verify the anti-proliferative effect of DCMI in lung CSCs, we treated LC1, LC2 and LC3 stem cells with different doses of DCMI and measured cell growth by quantifying the ATP content. As shown in Figure 2A, DCMI induced a significant reduction of lung CSCs growth and/or viability. Cancer stem cells are characterized by an increased activity of the Aldehyde deidrogenase (ALDH) and the quantification of ALDH activity is commonly utilized to evaluate the percentage of the stem cells in a certain cell population [31-33]. To verify whether DCMI has a preferential growth inhibitory action on the stem cell counterparts, we measured the ALDH activity (ALDEFLUOR) and found a significantly reduced content of ALDH-positive cells in DCMI-treated cells compared to the control cells (Figure 2B). These results suggest that the DCMI growth inhibitory activity is mainly ascribed to an effect on the stem cells population. In supporting of this statement, we also measured the sphere-forming capacity of lung CSC treated with DCMI. As shown in Figure 2C and 2D, DCMI treatment reduced both the number and the size of sphere-forming cells as compared to non-treated cells, thus confirming that DCMI negatively affects either the frequency of CSCs or their proliferation potential.

In tumor cell lines the anti-proliferative effect of DCMI has been associated with the blockade of the autophagic flux. To verify whether DCMI might exert similar effects on the authophagic machinery in lung CSCs, we measured the protein levels of the autophagosomal marker microtubule-associated protein light 1 chain 2 (LC3) and p62, a cargo protein that is degraded through the autophagic pathway [34, 35]. We observed that DCMI induces a significant increase of LC3 lipidation in a dose dependent manner without triggering p62 degradation (Figure 2E). The block of the autophagic flux is not accompanied by a significant induction of the caspase 3 activity, suggesting that DCMI exerts an anti-proliferative rather that a pro-apoptotic effect in lung CSCs. All together these findings indicate that DCMI inhibits the lung CSCs expansion through inhibition of their self-renewal and proliferation and these effects are associated with a deregulation of the autophagic flux.

### DCMI increases the cytotoxic effect of conventional chemotherapeutic drugs in lung CSCs

**Figure 3:**
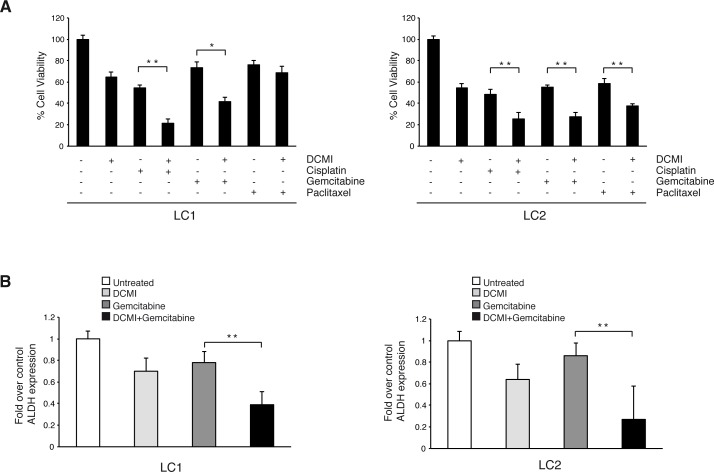
Combination of chemotherapy and DCMI increases cytotoxic effect on lung CSCs **A.** Viability of lung CSCs treated with 10 μM DCMI alone or in combination with 2.5 μg/ml cisplatin, 50 μM gemcitabine or 30 ng/ml paclitaxel for 48 h (LC1 left side; LC2 right side). Bars shown are the mean ± S.D. of three independent experiments. *P-value <0.05 **P-value <0.01. **B.** Fold variation of ALDEFLUOR-positive cells in treated lung CSCs with 10 μM DCMI alone or in combination with 2.5 μg/ml cisplatin, 50 μM gemcitabine or 30 ng/ml paclitaxel for 48 h as compared with vehicle-treated controls (LC1 left side; LC2 right side). Bars represent mean ± S.D.; **P-value <0.01 (*n* = 3).

Pharmacological inhibition of autophagy has been shown to enhance the anti-tumoral efficacy of different chemotherapeutic agents in cancer cells that have become chemoresistant [36-40]. To test whether DCMI could potentiate the cytotoxic effect of anti-neoplastic agents in lung CSCs, we treated LC1 and LC2 cells with cisplatin, gemcitabine or paclitaxel for 72 hours, in the presence or absence of 10 μm of DCMI and then measured the cell growth by quantifying the ATP content. As shown in Figure 3A and 3B, DCMI strongly sensitized lung CSCs to the toxic effect of the chemotherapeutic agents as assessed by the cell viability assay. We next verified whether DCMI together with gemcitabine might exert a preferential growth inhibitory action on the stem cells sub-population. To this aim, we measured the ALDH activity in LC1 and LC2 cells treated with gemcitabine alone or in combination with DCMI. We found that, while gemcitabine alone slightly reduces the percentage of ALDH-positive cells, the combination of DCMI and gemcitabine induced a significant reduced content of ALDH-positive cells. All together, these results suggested that DCMI treatment might be a valid method to increase the cytotoxic effect of conventional chemotherapeutic agents in lung CSCs.

### RNA-mediated silencing of ITCH impairs lung CSCs proliferation

**Figure 4:**
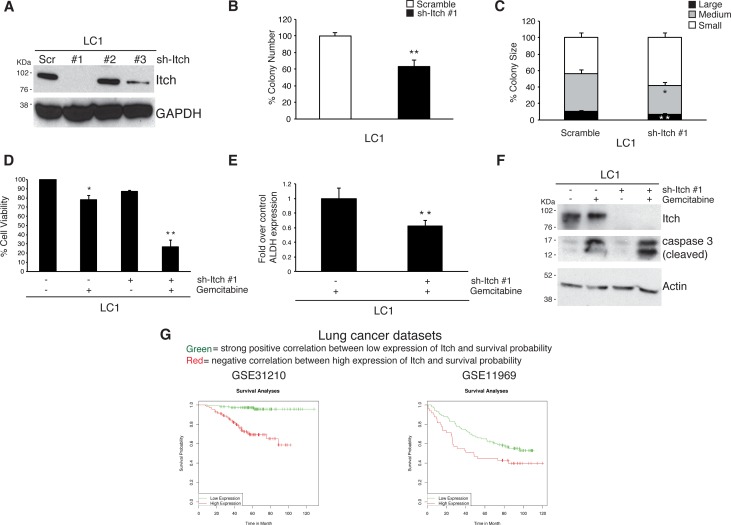
RNA-mediated silencing of Itch impairs lung CSCs proliferation **A.** Western blot analysis of Itch expression in lung CSC LC1 transfected with short-hairpin RNAs non-targeting (Scrambled) or direct against Itch (sh Itch). **B.** Colony formation in soft-agar culture of lung CSC LC1 transfected as above. Bars represent mean ± S.D.; **P-value <0.01 (*n* = 3). **C.** Size of colonies formed in soft-agar assay by lung CSC LC1 transfected as above. Bars represent mean ± S.D.; *P-value <0.05 and **P-value <0.01 (*n* = 3). **D.** Viability of lung CSC LC1 transfected as above and treated with 50 μM gemcitabine for 96 h. Bars represent mean ± S.D.; *P-value <0.05 and **P-value <0.01 (*n* = 3). **E.** Fold variation of ALDEFLUOR-positive cells in treated lung CSCs with 50 μM gemcitabine for 96 h. Bars represent mean ± S.D.; **P-value <0.01. **F.** Western blot analysis of lung CSC LC1 transfected as above and treated with 50 μM gemcitabine for 96 h. All whole cell extracts were analyzed by IB using antibodies to the indicated proteins. **G.** GEO lung adenocarcinoma data set (GSE31210 and GSE11969) were analyzed for the expression of Itch with computation estimation of Kaplan-Maier. Green line represents patients displaying high Itch expression levels while red line represents those with low expression. The R statistical package was used to perform survival analyses and to draw the KM plots.

Recently our group has identified DCMI as an inhibitor of the E3 ubiquitin ligase activity of Itch [22]. Similarly to DCMI, Itch silencing synergizes with anti-neoplastic agents in killing prostate, bladder and breast cancer cell lines. To verify whether Itch down-regulation affects lung CSCs homeostasis, we firstly tested three shRNA oligos for their efficiency to decrease Itch protein levels. We infected LC1 cells with lentiviral particles expressing shRNA oligos targeting different sequences of the Itch mRNA and identified sh-Itch #1 as the more efficient Itch targeting oligo (Figure 4A). Then, we analyzed the effect of Itch silencing on lung CSCs stemness properties and observed that Itch depletion decreases the sphere forming capability (Figure 4B and 4C). Importantly, we found that Itch silencing induces a marked increase of the pro-apoptotic effect of the gemcitabine, which is accompanied by a decrease of the ALDH positive cells, suggesting that, similarly to what observed in DCMI-treated cells, Itch silencing exerts a cytotoxic effect towards the stem cell counterpart (Figure 4D and 4E). At molecular level, we found that Itch depletion induces the activation of the apoptotic program in gemcitabine-treated cells, as reveled by the increase of the caspase 3 cleavage (Figure 4F). These results indicate that down modulation of Itch expression decreases the chemoresistance of lung CSCs and suggest that the expression levels of Itch might be predictive to evaluate the chemotherapeutic response and, as a consequence, the survival of the lung adenocarcinoma affected patients. To test this possibility, we assessed the impact of Itch expression levels on patient survival by performing a computational analysis in two publicly available lung adenocarcinoma primary tumor datasets [41]. We stratified the samples in two groups: patients displaying high Itch expression levels and those with low expression. Computation estimation of Kaplan-Maier in these two subgroups revealed that high levels of Itch negatively impact on patient survival (Figure 4G), indicating that Itch expression is a negative prognostic factor on patient survival and it might be functionally important to regulate the tumor progression.

## DISCUSSION

Despite the knowledge on lung cancer biology has significantly increased in the last twenty years, there have been limited progresses in the therapeutic management. However, the recent identification of CSCs as the cell population responsible for tumor initiation, propagation and resistance to therapy may provide an unprecedented tool to develop more effective treatments [42, 43]. Therefore, it is not surprising that many efforts have been concentrated to the identification and characterization of specific inhibitors of CSC homeostasis in order to attenuate their ability to survive conventional cytotoxic therapies and promote tumor recurrence [44-49]. In this report, we utilized as cellular model primary CSC cultures from three NSCLC surgical samples that showed the expected resistance to commonly used antineoplastic agents. We found that DCMI, the active metabolite of clomipramine, inhibits lung CSC growth, decreases their stemness potential and increases the cytotoxic effect of conventional chemotherapic agents.

Although we observed a slight increase of the caspase 3 cleavage upon DCMI treatment, this effect is not associated with PARP cleavage. It is possible that DCMI does not trigger a significant apoptotic signaling but rather it exerts a growth-suppressive effect due to its effect on the autophagic flux. Clomipramine and DCMI have previously described as inhibitors of the authophagic flux in several cancer cell lines [21]. This mechanism is preserved in lung CSCs. Although the role of autophagy in controlling cancer growth is controversial and it is likely to be tumor specific, many reports indicate that induction of autophagy upon nutrient, growth factor or oxygen deprivations, functions to maintain the survival of tumors cells [9, 11, 50, 51]. Furthermore, many chemotherapeutic agents induce autophagy, most likely by causing damage to DNA, cellular proteins, and organelles [51, 52]. So it is reasonable that inhibition of the autophagy program might be a valid method to sensitize cells to endogenous or exogenous stresses. Accordingly, inhibitors of autophagy augment the efficacy of anticancer agents in many preclinical models, indicating that autophagy might be utilized by the cells to sustain cancer cells against the effect of cytotoxic compounds, ensuring thus the survival of transformed cells [11, 36, 37, 52]. In agreement with these observations, autophagy inhibition with hydroxychloroquine in combination with anticancer regiments is currently in clinical trial for patients with several refractory malignancies, including prostate, lung, breast and brain tumors. In this scenario, we characterized the effects of the DCMI-mediated autophagy inhibition in lung CSCs and showed that DCMI treatment inhibits the stemness potential of lung CSCs and potentiates the anti-tumoral effects of conventional chemotherapic agents.

Besides its effect as inhibitor of the autophagic flux, DCMI has been recently reported as an inhibitor of the E3 ubiquitin ligase ITCH [22]. Itch belongs to the HECT-type E3 ubiquitin ligase and controls the proteasome-dependent degradation of several proteins involved in the regulation of cell survival, cell growth and inflammatory response. Among the substrates of Itch, the transcription factors p73 and p63 are particularly important for their involvement in the regulation of cell survival and proliferation [24, 53]. p73 is a structural and functional homologue of the tumour suppressing transcription factor p53 [54-57]. p53 is considered the guardian of the genome since it is able to restrict cell proliferation or induce DNA repair in cells exposed to different cellular stresses avoiding that DNA damage is converted to inherited mutation [58-60]. A variety of intracellular and exogenous stimuli are indeed able to stabilize and activate p53 activity towards a large number of transcriptional targets, including micro-RNAs, pro-survival, pro-apoptotic, cell cycle and metabolic genes [61-76]. Based on its important role in mediating the cellular response to various stimuli, it is not surprising that deregulation of its activity is strictly associated with the onset and the progression of pathological processes [77-79], mainly tumor development [80-82]. The most significant evidence involving p53 in cancer is its high mutation rate in cancers as well as its ability, when mutated, to drive cancer metastasis [83-89]. Several novel drug-design or high content screening is attempting to use the p53 pathway for therapeutic application in cancer. The majority of the drugs targeting p53 pathway are exploited to induce p53 protein stabilization rather than activate its transcriptional activity [90-92]. However, more than 50% of human tumors harbor mutations in the p53 gene. Therefore, drugs regulating the p53-independent apoptotic pathways would be extremely useful to restrict cell proliferation in the p53 defective tumors. p73, similarly to p53, is able to mediate cell cycle arrest and apoptosis in response to DNA damage-induced cellular stress [56]. p73 is rarely mutated in cancer and it is expressed at different protein isoforms, exhibiting contrasting effects on cell tumor development [55, 93, 94]. Specifically the TAp73 isoforms mimics the tumor suppressive function of p53 and its expression is maintained at low levels in mammalian cells by different ubiquitin-dependent mechanisms, among them the Itch-dependent degradation of p73 is the most characterized [28]. Therefore, inhibitors of Itch activity might be useful to activate p73-dependent apoptotic program in those tumors harbouring p53 mutations. Although we do not know whether the DCMI-mediated effect on lung CSCs survival depends of its activity as inhibitor of autophagic flux or inhibitor of Itch, we reported that the shRNA-mediated inhibition of Itch expression potentiates the anti-apoptotic effects of gemcitabine in lung CSCs, similarly to what we observed in DCM1-treated cells. Furthermore, we found that depletion of Itch decreases the stemness capability of lung CSCs, as measured by clonogenicity and ALDH activity assay. These results are in agreement with several reports demonstrating that Itch is required for both embryonic stem cell (ESC) self-renewal capacity and somatic cell reprogramming efficiency, through its control on Oct-4 protein stability [95]. Moreover, while the DCMI-mediated inhibition of Itch activity occurs at high micromolar concentration *in vitro* [22], its effect on cancer cell growth is evident at low micromolar concentration, suggesting that DCMI might block cancer cell growth in an Itch-independent manner.

We have also reported data showing that Itch expression level is a negative prognostic factor on patient survival in two lung adenocarcinoma primary tumor datasets. This bioinformatic analysis suggests that Itch levels might be predictive to establish an efficient chemotherapeutic response of lung tumors. Furthermore, this analysis also suggests the proof of principle to concentrate our efforts in identifying and characterizing more potent and specific inhibitors of Itch. Indeed, although the proteasome inhibitor Bortezomib is clinically utilized to treat patients with multiple myeloma or mantle-cell lymphoma, it is not curative and toxic in solid malignancies, probably due to its broad biological response [96-98]. Recently, preclinical studies demonstrated that autophagy inhibition by hydroxychloroquine augments the efficacy of the proteasome inhibitor bortezomib in myeloma, indicating that the combination of autophagy and proteasome inhibition might be clinically useful for improving the outcomes of this neoplasia [99, 100]. Thus targeting specific E3 ubiquitin ligases might represent a potentially more effective therapeutic strategy, limiting unwanted side effects.

In conclusion, although the mode of action of DCMI should be further clarified, our data demonstrated for the first time that DCMI treatment might be a valid approach to regulate lung CSC homeostasis and their response to chemotherapeutic agents, supporting a potential clinical application.

## MATERIALS AND METHODS

### Antibodies and reagents

Gemcitabine, Paclitaxel and Cisplatin were purchased from Sigma-Aldrich (St. Louis, MO, USA). ALDEFLUOR assay was from StemCell Technologies (Durham, NC, USA). CD133/1PE (used for flow cytometry) was from Miltenyi Biotec (Bergisch Gladbach, Germany). Monoclonal antibodies: anti-ITCH antibody was from BD Transducion Laboratories (San Jose, CA, USA), anti-p62 SQSTMI from Santa Cruz Biotechnologies (Dallas, Texas, USA), anti-PARP1 from ENZO (New York, NY, USA), anti-actin from Sigma-Aldrich (St. Louis, MO, USA); polyclonal antibodies: anti-LC3 was purchased from Sigma-Aldrich and anti-caspase3 from Cell Signaling (Danvers, MA, USA). Secondary anti-mouse and anti-rabbit antibodies coupled to horseradish peroxidase were from Bio-Rad (Hercules, CA, USA).

### Cell cultures

Lung CSCs were isolated as previously described [6] from surgically resected tumor samples through selective culture in serum-free medium containing EGF 20 ng/ml and basic FGF 10 ng/ml (PeproTech, London, UK) at 37 C° with 5% CO_2_. Non-treated flasks for tissue culture (Corning, Tewksbury, MA, USA) were used to reduce cell adherence and support growth of lung CSCs as multicellular spheres. The medium was replaced twice a week until cells started to grow forming floating aggregates. Cultures were expanded by mechanical dissociation of spheres, followed by re-plating of both single cells and residual small aggregates in complete fresh medium. Lung CSCs differentiation was obtained by culture overnight in Dulbecco's modified Eagle's medium (DMEM) supplemented with 10% fetal bovine serum (FBS) (Gibco, Invitrogen, Carlsbad, CA, USA) and for additional 3 days in Bronchial Epithelial Cell Growth Medium (Cambrex, East Rutherford, NJ, USA).

### Viability assay

Lung CSCs viability upon treatment with chemotherapeutic drugs, or DCMI was determined with the CellTiter-Glo assay (Promega, Madison, WI, USA) according to the manufacturer's instructions. In brief, 1.5 × 10^3^ dissociated lung CSCs were plated in triplicate in 96-well flat bottom plates. Chemoterapeutic agents were added at the following final concentration: gemcitabine 50 uM, paclitaxel 30ng/ml, cisplatin 5 ug/ml. Cell viability was analyzed after 72 hours with a Promega Glomax Multi Detection System plate reader (Promega, Madison, WI, USA).

### Sphere-forming ability assay

The sphere-forming capacity of lung CSCs was carried out by plating 500 cells per well in triplicate in 24-well plates containing a soft agar bilayer (0.3% top and 0.4% bottom layer, SeaPlaque Agarose, Cambrex) with or without DMCI. Cultures were incubated at 37 C° for 21 days. Colonies were stained with crystal violet (0.01% in 10% methanol). Data shown represent the percentage of colonies normalized to the number of plated cells.

### Western blotting

Immunoblot analysis was performed as previously described [101]. Briefly, whole cell extracts were obtained by lysing cell pellets with Triton Buffer (50 mM Tris-Hcl pH 7.5, 250 mM NaCl, 50 mM NaF, 1mM EDTA 1 pH 8, 0.1% Triton), supplemented with protease and phosphatase inhibitors. Lysate concentrations were determined by the Bradford assay (Bio-Rad Laboratories). Proteins were separated by SDS-PAGE, transferred onto PVDF membranes and blocked with PBS-T (Phosphate-buffered saline and 0,1%Tween-20) containing 5% non-fat dry milk for one hour at room temperature (RT). The incubation with primary antibodies was performed for two hours at RT, followed by incubation with the appropriate horseradish peroxidase-conjugated secondary antibody. Detection was performed with ECL Western Blot Reagent (Perkin Elmer) or with Super SignalWest Pico (Pierce).

### RNA interference

Briefly, cells were seeded at a density of 1.5 × 10^6^ cells/well in a 6-well plates and incubated over night at 37 C° in the presence of SMART Vector 2.0 Lentiviral shRNA particles (FE5S00500001 non targeting Human siRNA, FE5SK0071960010 Human Itch Dharmacon/Thermo Scientific, Lafayette, CO, USA) according to manufacturer's instructions. Transduced GFP-positive cells were determined by fluorescent microscopy 48 h after infection. Cells were collected and lysates were analyzed for protein expression.

### Flow cytometry analysis

Expression of lung CSCs marker was evaluated by flow cytometry. Lung CSCs were dissociated as a single cells, washed in PBS and incubated with the appropriate dilutions of control or specific antibodies for 1 hour at room temperature. Cells were stained live in the staining solution containing BSA and PE-conjugated monoclonal anti-CD133 (clone AC133 Miltenyi Biotech) at the concentration recommended by the manufacturers. Corresponding isotype-matched mouse immunoglobulins were used as negative controls (BD Bioscience). Lung CSCs were dissociated as single cells, washed in PBS and incubated with the appropriate diluitions of control or specific antibodies for 1 hour at room temperature. Fluorescence intensity of labeled cells was evaluated by FACS Calibur (Becton Dickinson, Franklin Lakes, NJ, USA). Ten thousand events were evaluated using the Cell Quest (BD, Franklin Lakes, NJ) and Modfit LT (Verity Software, BD) programs.

### Aldefluor assay

The Aldefluor kit assay (StemCell Technologies, Vancouver, Canada) was used to profile the aldheyde dehydrogenase (ALDH) activity in lung CSCs. Cells were incubated in Aldefluor assay buffer containing the ALDH protein substrate BODIPY-aminoacetaldehyde (BAAA) for 40 min at 37 C°. Cells that could catalyze BAAA to its fluorescent product BODIPY-aminoacetate (BAA) were considered ALDH^+^. Sorting gates for FACS were drawn relative to cells baseline fluorescence, which was determined by the addition of the ALDH specific inhibitor diethylaminobenzaldehyde (DEAB) during the incubation and DEAB-treated samples served as negative controls. Cells were sorted by a FACS Calibur (Becton Dickinson, Franklin Lakes, NJ, USA).

### Statistical and data analysis

All data are presented as mean ± standard deviation (S.D.). Statistical significance was determined by ANOVA. And threshold was set at 0.05. A *P*-value < 0.05 is represented by a single asterisk, a *P* value <0.01 is represented by a double asterisk.

Human lung adenocarcinoma sample data was downloaded from the GEO database, accession numbers GSE31210 (226 patients) and GSE11969 (149 patients). The analysis of the Kaplan-Maier estimation curves was performed utilizing the PPISURV bioinformatics tool (http://bioprofiling.de/Results/PPISURV_1290_1434547360/main.html) as previously described [41]. Briefly, the separation of patients into “cohort 1” and “cohort 2” along with the survival information was used to identify any significant differences in the survival outcome. The R statistical package was used to perform survival analyses and to draw the KM plots.

## Abbreviations

CSCs: cancer stem cells
DCMI: desmethylclomipramine
ITCH: HECT ubiquitin E3 ligase
EGF: epidermal growth factor
b-FGF: basic fibroblast growth factor
EGFR: epidermal growth factor receptor
KRAS: Kirsten rat sarcoma
